# Egg-Clutch Biomechanics Affect Escape-Hatching Behavior and Performance

**DOI:** 10.1093/iob/obae006

**Published:** 2024-03-13

**Authors:** B A Güell, J G McDaniel, K M Warkentin

**Affiliations:** Department of Biology, Boston University, Boston, MA 02215, USA; Department of Mechanical Engineering, Boston University, Boston, MA 02215, USA; Department of Biology, Boston University, Boston, MA 02215, USA; Gamboa Laboratory, Smithsonian Tropical Research Institute, Apartado Postal 0843-03092, Panamá, República de Panamá

## Abstract

Arboreal embryos of phyllomedusine treefrogs hatch prematurely to escape snake predation, cued by vibrations in their egg clutches during attacks. However, escape success varies between species, from ∼77% in *Agalychnis callidryas* to just ∼9% in *A. spurrelli* at 1 day premature. Both species begin responding to snake attacks at similar developmental stages, when vestibular mechanosensory function begins, suggesting that sensory ability does not limit the hatching response in *A. spurrelli. Agalychnis callidryas* clutches are thick and gelatinous, while *A. spurrelli* clutches are thinner and stiffer. We hypothesized that this structural difference alters the egg motion excited by attacks. Since vibrations excited by snakes must propagate through clutches to reach embryos, we hypothesized that the species difference in attack-induced hatching may reflect effects of clutch biomechanics on the cues available to embryos. Mechanics predicts that thinner, stiffer structures have higher free vibration frequencies, greater spatial attenuation, and faster vibration damping than thicker, more flexible structures. We assessed clutch biomechanics by embedding small accelerometers in clutches of both species and recording vibrations during standardized excitation tests at two distances from the accelerometer. Analyses of recorded vibrations showed that *A. spurrelli* clutches have higher free vibration frequencies and greater vibration damping than *A. callidryas* clutches. Higher frequencies elicit less hatching in *A. callidryas*, and greater damping could reduce the amount of vibration embryos can perceive. To directly test if clutch structure affects escape success in snake attacks, we transplanted *A. spurrelli* eggs into *A. callidryas* clutches and compared their escape rates with untransplanted, age-matched conspecific controls. We also performed reciprocal transplantation of eggs between pairs of *A. callidryas* clutches as a method control. Transplanting *A. spurrelli* embryos into *A. callidryas* clutches nearly tripled their escape success (44%) compared to conspecific controls (15%), whereas transplanting *A. callidryas* embryos into different *A. callidryas* clutches only increased escape success by 10%. At hatching competence, *A. callidryas* eggs are no longer jelly-encapsulated, while *A. spurrelli* eggs retain their jelly coat. Therefore, we compared the hatching response and latency of *A. spurrelli* in de-jellied eggs and their control, jelly-encapsulated siblings using manual egg-jiggling to simulate predation cues. Embryos in de-jellied eggs were more likely to hatch and hatched faster than control siblings. Together, our results suggest that the properties of parentally produced egg-clutch structures, including their vibration biomechanics, constrain the information available to *A. spurrelli* embryos and contribute to interspecific differences in hatching responses to predator attacks.

## Abstract (es)

**Resumen** Los embriones arbóreos de las ranas Phyllomedusinae eclosionan prematuramente para escapar de la depredación de las serpientes, señaladas por las vibraciones en sus posturas durante los ataques. El éxito de escape varía entre especies, desde ∼77% en *Agalychnis callidryas* hasta sólo ∼9% en *A. spurrelli* a un día antes de la eclosión espontanea. Ambas especies comienzan a responder a los ataques de serpientes en etapas del desarrollo similares cuando inicia la función mecanosensorial vestibular, lo que sugiere que la capacidad sensorial no limita la respuesta de eclosión en *A. spurrelli*. Estructuralmente, las posturas de *A. callidryas* son gruesas y gelatinosas, mientras que las de *A. spurrelli* son más delgadas y rígidas. Esta diferencia en estructura de la postura podría afectar su movimiento y la propagación de las señales de vibración que perciben los embriones durante los ataques de serpientes. Por lo tanto, investigamos la hipótesis de que las diferencias entre especies en las respuestas de eclosión a los ataques de serpientes se deben a la influencia de la biomecánica de las posturas en las señales disponibles para los embriones. Mecánicamente, las estructuras más delgadas deberían tener frecuencias de resonancia más altas, mayor atenuación espacial y una amortiguación de vibraciones más rápida que las estructuras más gruesas y flexibles. Evaluamos la biomecánica de las posturas mediante la incorporación de acelerómetros pequeños dentro de las posturas de ambas especies, así como la grabación de vibraciones causadas por pruebas de excitación estandarizadas a diferentes distancias del acelerómetro. Los análisis de las vibraciones registradas indican que las posturas de *A. spurrelli* tienen frecuencias de vibración más altas y una mayor amortiguación de vibraciones que las posturas de *A. callidryas*. Las frecuencias más altas provocan menos eclosión en *A. callidryas*, y una mayor amortiguación podría reducir la cantidad de vibración que pueden percibir los embriones. Para probar directamente si la estructura de posturas afecta el éxito de escape en los ataques de serpientes, trasplantamos huevos de *A. spurrelli* en posturas de *A. callidryas* y comparamos sus tasas de escape con controles conespecíficos de la misma edad no trasplantados. También realizamos trasplante recíproco en *A. callidryas* como control de método. El trasplante de embriones de *A. spurrelli* en posturas de *A. callidryas* casi triplicó su éxito de escape (44%) en comparación con los controles conespecíficos (15%), mientras que el trasplante de huevos de *A. callidryas* aumentó el éxito de escape en solo un 10%. Al momento de la eclosión, los huevos de *A. callidryas* ya no están encapsulados en gelatina, mientras que los huevos de *A. spurrelli* conservan su cápsula de gelatina. Por lo tanto, comparamos la respuesta de eclosión y la latencia de *A. spurrelli* en huevos sin gelatina y sus hermanos de control, encapsulados en gelatina, usando movimientos manuales de huevos para simular señales de depredación. Los embriones en huevos sin gelatina tenían más probabilidades de eclosionar y eclosionaron más rápido que los hermanos de control. Nuestros resultados sugieren que las propiedades de las estructuras producidas por padres, como las posturas de huevos, incluida su biomecánica de vibración, restringen la información disponible para los embriones de *A. spurrelli* y contribuyen a las diferencias interespecíficas en las respuestas de eclosión a los ataques de los depredadores.

## Introduction

Substrate-borne vibrations are ubiquitous and often serve as a source of information and a mode of communication. Animals from a broad range of taxa, from worms and insects to fishes and mammals, use signals and incidental cues that propagate from sources to receivers as vibrations traveling through substrates (Hill 2008,^ 2009^). How such behaviorally relevant vibrational information is transmitted and received depends strongly on the characteristics of the transmission medium. Different substrates have different attenuation and resonance properties that alter the structure of signals, often imposing constraints on information transmission (Hill 2008; Cocroft et al. 2014; Hill et al. 2019). Thus, to understand why some animals effectively use substrate-borne vibrations to cue adaptive behavioral responses while others, in apparently similar contexts, respond weakly or not at all, it is essential to characterize how vibrations propagate through those animals’ immediate environments.

Hatching in response to mechanosensory cues, particularly vibrations but also direct contact cues and perhaps other elements of physical disturbance, is widespread (Warkentin 2011a; Warkentin et al. 2022). Many animals including invertebrates (Whittington and Kearn 1988,  2011; Mukai et al. 2012, 2014; Nishide and Tanaka 2016; Endo et al. 2019), fishes (Martin et al. 2011), amphibians (Warkentin 1995, 2000, 2005, 2011b; Buckley et al. 2005; Gomez-Mestre et al. 2008; Touchon et al. 2011), and reptiles (Doody 2011; Doody et al. 2012; Doody and Paull 2013) use disturbances of eggs as hatching cues. Some embryos respond to stereotyped vibrational signals from their parents, but most hatch in response to more variable incidental cues produced by abiotic sources, siblings, hosts, and predators (reviewed in Warkentin et al. 2022). Regardless of their source, the characteristics of vibrational cues depend strongly on the mechanical properties of the substrates through which vibrations travel (Bacher et al. 1997; Casas et al. 1997; Magal et al. 2000). The mechanosensory cues known to stimulate hatching largely result from direct physical disturbances to eggs or egg clutches, rather than from vibrations transmitted over long distances through substrates (Warkentin et al. 2022). Moreover, all vibrational cues detected by embryos within their eggs must first excite and propagate through these structures. Thus, the vibrational cues available to embryos largely depend on the vibration mechanics of parentally produced structures, including egg capsules, clutches, and nests.

Egg-clutch structure is known to affect embryo fitness in several contexts including hypoxia, dehydration, and invertebrate predation (Warkentin et al. 2006a; Strathmann and Strathmann 1989; Cohen and Strathmann 1996; Strathmann and Hess 1999; Touchon and Warkentin 2009; Delia et al. 2020). However, the role of egg-clutch structure in mediating vibrational information transfer has only been considered in one species: the red-eyed treefrog (*Agalychnis callidryas*) (Caldwell 2010). Caldwell (2010) characterized the free vibration dynamics and vibration transmission properties of *A. callidryas* egg clutches. This work demonstrated that most free vibrations in *A. callidryas* clutches—i.e., those that occur after an object is disturbed and then left to move freely without any external forcing—are below 50 Hz. Moreover, high frequencies (>200 Hz) attenuate rapidly. This work suggests that egg-clutch properties strongly influence the vibrational cues available to embryos (Caldwell 2010). Whether and how interspecific differences in egg-clutch structure affect the vibrational environment of embryos and their escape-hatching behavior remains unknown. Here, we examine how vibrations propagate through two different types of treefrog egg clutches and assess the effect of individual egg and whole egg-clutch structure on embryo escape-hatching behavior.

Frogs in the subfamily Phyllomedusinae (Anura: Hylidae) offer an excellent opportunity to investigate the effects of egg-clutch structure on vibration properties and embryo behavior. Phyllomedusines are arboreal frogs that lay terrestrial egg clutches on plants that overhang rainforest ponds (Duellman 1968,  1970). Embryos of all tested species are capable of hatching early in response to hypoxia cues when flooded and mechanosensory cues produced by snake attacks (Gomez-Mestre et al. 2008). However, escape rates in response to snake attacks vary substantially among species, from 77% escape success in *A. callidryas* to 9% in *A. spurrelli* at 1 day premature (Gomez-Mestre and Warkentin 2007; Gomez-Mestre et al. 2008). Video recordings of predation by cat-eyed snakes (*Leptodeira ornata*; formerly *L. septentrionalis*, Barrio-Amorós 2019; Torres-Carvajal et al. 2020) on *A. spurrelli* clutches show that, unlike those of *A. callidryas, A. spurrelli* eggs are minimally displaced during attacks (BAG and KMW personal observation). This suggests that their egg-clutch structure may limit vibrational cues available to *A. spurrelli* embryos and thus contribute to their low escape success.

**Fig. 1:**
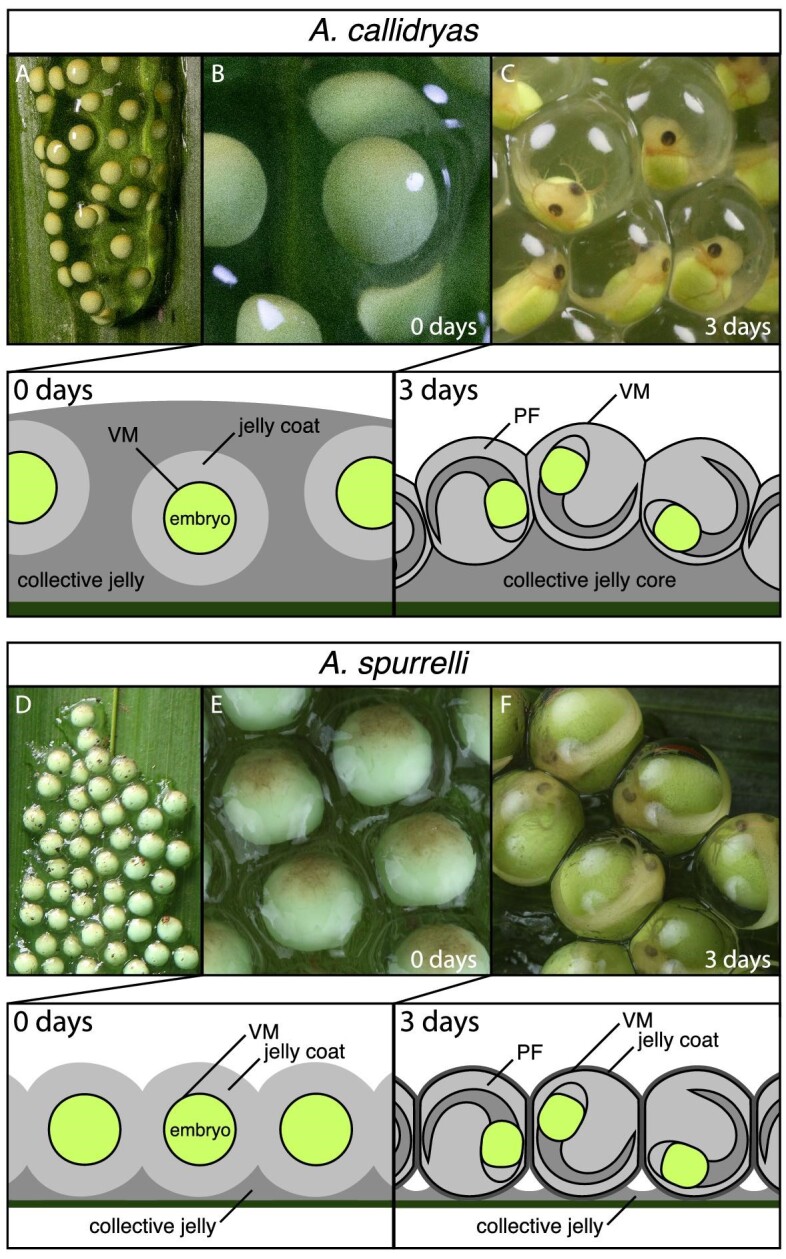
Ontogeny of *A. spurrelli* and *A. callidryas* egg-clutch and egg-capsule structure. Photos and illustrations show changes to clutch and capsule structure from age 0 to 3 days. Clutch and egg morphology is relatively stable from age 3 days until hatching in both species. *Agalychnis callidryas* clutches are highly gelatinous at oviposition (0 days) (**A**), and individually jelly-coated eggs float within ample collective jelly (**B**). As *A. callidryas* embryos develop, their perivitelline chamber expands, their jelly coat stretches, thins, and loses integrity, and eggs move outwards to surround a collective jelly core (**C**). At oviposition (0 days) *A. spurrelli* clutches form a thin monolayer of eggs (**D**) with thick, rubbery jelly coats and little collective jelly (**E**). The jelly coat thins and hardens as embryos develop and the perivitelline chamber swells (**F**). Illustrations are profile views of eggs within panels B and C for *A. callidryas* and E and F for *A. spurrelli.* VM: vitelline membrane, PF: perivitelline fluid.

Most phyllomedusine treefrogs, including *A. callidryas*, lay highly gelatinous egg clutches in which individual eggs are initially embedded within a collective mass of jelly (Pyburn 1963, 1970; Duellman 1968, 2001) (Fig. 1A). Each egg includes the fertilized ovum closely surrounded by the vitelline membrane (i.e., oocyte coat), which in turn is enclosed in a thick outer jelly coat (*sensu*  Shu et al. 2015; also see Altig and McDiarmid 2007; and Delia et al. 2020 for a review of egg-clutch structure in glassfrogs; Fig. 1). In *A. callidryas*, as embryos develop the perivitelline space within the vitelline membrane swells and the jelly coat stretches and thins, losing integrity. Concurrently, the eggs move to the outside of their collective jelly mass (Fig. 1A–C). By 3 days, the closely apposed eggs encase a core of collective jelly, with little or no jelly between the vitelline membrane and air (Fig. 1C). In contrast, the egg clutches of *A. spurrelli* (and those of *A. saltator*; see Roberts 1994) contain very little collective jelly at oviposition and never develop a gelatinous jelly core (Fig. 1D–F). Unlike most phyllomedusines, female *A. spurrelli* do not absorb water into their bladders and use it to hydrate egg jelly during oviposition (Pyburn 1970; Faivovich et al. 2010; Güell et al. 2019). Instead, their clutches are laid in a thin monolayer that is only slightly thicker than the diameter of individual eggs, whose jelly coats are tough and sticky (Gomez-Mestre and Warkentin 2007; Bland 2013) (Fig. 1D–F). As *A. spurrelli* embryos develop and their perivitelline space swells, the jelly coat thins but retains its integrity, forming a strong, continuous rubbery layer around the perivitelline membrane (Fig. 1F). The clutch structure remains flat, with minimal collective jelly between eggs and leaf (Fig. 1F). The ecological and evolutionary significance of these structural differences among phyllomedusine egg clutches are unclear. However, they are also associated with differences in adult reproductive strategies and rates of embryonic development (Gomez-Mestre and Warkentin 2007; Gomez-Mestre et al. 2008; Güell and Warkentin 2023b).

We recently found that *A. spurrelli* and *A. callidryas* begin hatching in response to mechanosensory cues at similar developmental stages, at the onset of vestibular mechanosensory function, suggesting that the interspecific variation in escape success is not due to sensory or physiological constraints in *A. spurrelli* (Güell and Warkentin 2023b). Instead, variation in escape rates may be due to differences in the vibration mechanics of different types of egg clutches. Escape hatching by *A. callidryas* during snake attacks is cued by vibrations excited in clutches (Warkentin 2005), and embryos use multiple vibration properties, such as frequency and temporal pattern, to assess risk (Warkentin 2005; Warkentin et al. 2006b; Jung et al. 2022). For example, vibrations excited by predator attacks are dominated by low frequencies, characteristic of clutch free vibrations (Caldwell et al. 2009, 2010). In vibration playback experiments, *A. callidryas* embryos hatch more in response to low frequencies, especially <50 Hz, but not to higher vibration frequencies (Caldwell et al. 2009). Rainstorms also excite intense egg-clutch vibrations and are a common type of disturbance, but they do not elicit escape hatching. Vibrations excited by rain have a broader frequency range than do snake attack vibrations, including higher frequencies that, in playbacks, reduce embryos’ response to concurrently presented low frequencies (Warkentin 2005; Caldwell et al. 2009, 2010).

We hypothesized that differences between *A. spurrelli* and *A. callidryas* in vibration biomechanics of clutch structures explain, at least in part, their different escape success in response to snake attacks. We specifically hypothesized that three aspects of egg-clutch biomechanics would differ between species: (1) free vibration frequencies, (2) vibration decay over space, and (3) vibration decay over time. The vibrations of an object during the application of a force, i.e., forced vibrations, vary substantially with the properties of the force applied (Ginsberg and Seemann 2001). Free vibrations occur when substrates vibrate with no external force applied to them, i.e., following a forcing event, and depend strongly on the properties of the object itself (Snowdon 1968; Ginsberg and Seemann 2001). In general, the free vibrations of dynamic structures with viscous damping—such as gelatinous egg clutches—are characterized by an exponential decay of the oscillation. These free vibration frequencies (also known as natural or resonant frequencies) are typically measured experimentally in the frequency domain by determining the rate of vibration oscillation and decay following an impact on the structure. Analytical methods for the study of the free-vibration properties of a structure, known as modal analysis (Fu and He 2001), are well developed in mechanical engineering, and have been adapted to test *A. callidryas* egg clutches in the field (Caldwell 2010). Tested structures are typically fabricated of uniform materials with precise dimensions, resulting in highly precise and accurate measurements of mechanical properties. However, most structures through which biologically relevant vibrations propagate are structurally complex, variable in shape and size, and often respond nonlinearly to vibrational forces (Michelsen et al. 1982; De Langre 2008; Hill 2009; Hill et al. 2019; Roberts and Laidre 2019). The highly variable and irregular structure of *Agalychnis* egg clutches paired with the considerable variation in oviposition substrates (e.g., leaves, branches, moss, etc.) make analyzing their biomechanical properties using traditional methods of modal analysis difficult. Thus, we assessed the vibration biomechanics of *Agalychnis* egg clutches in the field using two kinds of standardized excitation tests, largely following methods developed by Caldwell (2010). We then developed methods to analyze the free vibration properties of egg clutches in the time domain.

Based on the stiffer, thinner structure of *A. spurrelli* clutches versus the more gelatinous, thicker structure of *A. callidryas* clutches, we predicted that *A. spurrelli* would have higher free vibration frequencies as well as greater vibrational damping over space and time, compared to *A. callidryas*. Based on the response of *A. callidryas* embryos, higher vibration frequencies in *A. spurrelli* could also reduce hatching (Caldwell et al. 2009, 2010). Greater attenuation of vibrations traveling across a clutch would reduce the vibrational stimulation to embryos farther from the snake, potentially reducing escape hatching. Moreover, faster decay rates would reduce vibration durations and alter temporal patterns, which might also alter embryo responses (Warkentin et al. 2006b). To test our predictions, we embedded small accelerometers within *A. callidryas* and *A. spurrelli* egg clutches and performed standardized excitation tests to compare their vibration mechanics. Then, to assess the impact of egg-clutch structure on escape-hatching success more directly, we conducted snake predation experiments with *A. spurrelli* eggs transplanted into *A. callidryas* egg clutches, compared with *A. spurrelli* in their own clutches. If the structure of *A. callidryas* clutches more effectively transmits vibrational cues to risk, the escape success of *A. spurrelli* embryos in attacks should improve with transplantation into *A. callidryas* clutches.

The properties of individual egg capsules may also affect escape-hatching success in *A. spurrelli*. In prior work across the onset of hatching competence, the youngest *A. spurrelli* embryos responding to both hypoxia and mechanosensory cues were often stalled by the jelly coat, prolonging the time they spent in a state of body compression, part way through the vitelline membrane (Güell and Warkentin 2023b). Such a delay in hatching, or even complete failure to hatch, might contribute to the lower escape-hatching success of more developed *A. spurrelli* embryos as well, if egg-capsule structure compromises hatching speed or success during snake attacks. Thus, to assess the impact of egg-capsule structure on escape-hatching performance, we used standardized egg-jiggling as a simulated attack cue (Güell et al. 2022) to assess the hatching response and latency of embryos in de-jellied (i.e., removed from their jelly coats) and control *A. spurrelli* eggs. We predicted de-jellying would increase the response rates and escape speeds of *A. spurrelli* embryos.

## Materials and methods

### Egg-clutch collection and care

We collected recently laid *A. spurrelli* and *A. callidryas* egg clutches from Shampoo Pond (8°24′55′′N, 83°20′45′′W) on Costa Rica's Osa Peninsula and brought them to an ambient temperature and humidity laboratory at Osa Conservation's Piro Biological Station. We taped leaves with clutches onto plastic cards with the long axis of the clutch oriented vertically, as is common in nature, then placed them in cups above aged tap water within large plastic bins with partially screened lids (egg humidors). We removed any dead or unfertilized eggs at setup and misted egg clutches and humidors frequently with rainwater to ensure healthy levels of egg and clutch hydration (Salica et al. 2017; González et al. 2021). *Agalychnis callidryas* mostly lays eggs between 10:00 and 2:00 h (Warkentin 2002; Warkentin et al. 2005), and *A. spurrelli* mostly lays eggs between midnight and 6:00 h (BAG personal observation). Since we did not know oviposition times for individual clutches, for ease of comparison we assigned embryo ages for both species starting from midnight of their oviposition night (Warkentin 2002; Warkentin et al. 2005). This likely overestimates *A. spurrelli* ages by a few hours; however, since we were mainly interested in comparing escape rates within species, not across them, this is not an issue. We returned all hatched tadpoles to their pond following our experiments. This research was conducted under BU IACUC protocol 18–003 and permits from the Costa Rican Ministerio de Ambiente y Energía (MINAE) and the Sistema Nacional de Áreas de Conservación (SINAC) (ACOSA-INV-048–18, ACOSA-INV-033–19, and SINAC-ACOSA-DASP-PI-R-019–2021).

### Excitation tests and vibration recordings from egg clutches

Between August 13–20, 2019, we performed a series of standardized mechanical excitation tests on egg clutches of *A. callidryas* (*N* = 17) and *A. spurrelli* (*N* = 16) to assess their vibration responses. Early in development, the perivitelline space of the egg expands in both species, and *A. callidryas* clutches change from smooth-surfaced globs with small embedded eggs to bunches of larger eggs around jelly cores (Fig. 1); however, in both species the overall morphology of clutches remains relatively stable from age 3 days until hatching (Pyburn 1963; Cohen et al. 2019; González et al. 2021; BAG personal observation). In *A. callidryas*, mechanical testing of clutches found similar vibrational properties at ages 3–5 days (Caldwell 2010), although in behavioral tests embryo responses to vibrations change from absent to strong over this period (Warkentin et al. 2017; Jung et al. 2020; Güell et al. 2022). Embryos of *A. spurrelli* reach mechanoresponsive developmental stages and hatch spontaneously almost a full day earlier than *A. callidryas* embryos (Güell and Warkentin 2023b). To assess biomechanical differences relevant to embryo behavior while avoiding structural changes caused by hatching, we tested clutches of both species at age 3 days, after the initial change in clutch morphology but before the onset of mechanosensory-cued hatching (Warkentin et al. 2017; Güell and Warkentin 2023b).

To record vibrations from egg clutches, we embedded a small accelerometer (0.14 g, AP19, AP Technology International, Oosterhout, The Netherlands) into the jelly of each clutch shortly before testing, following established methods (Warkentin 2005; Caldwell et al. 2009; Caldwell 2010; Warkentin et al. 2022). We used a single accelerometer for all vibration recordings. To keep the accelerometer dry, we potted it in a thin layer of silicone sealant. To standardize vibration recordings, we placed the accelerometer between the middle and lower third of each clutch, leaving room above it for impact testing at two distances, and we aligned its axis of sensitivity with the vertical axis of each clutch, parallel to gravity when tested. In *A. callidryas*, we used forceps to create an opening in the collective jelly from the side of each clutch, inserting the accelerometer through the jelly to rest among the eggs (Movie S1). Since the clutches of *A. spurrelli* lack an extensive gelatinous core (Fig. 1), we removed one egg per clutch and inserted the accelerometer in its place (Movie S2). Vibration signals from the accelerometer were processed by a signal conditioner (Type 1704, Brüel & Kjær, Virum, Denmark), digitized with an external sound card (Scarlett 2i2, Focusrite, High Wycombe, UK), and recorded using Raven Pro (Cornell Lab of Ornithology 2022) at 44.1 kHz on a MacBook Pro. All recording components were battery powered to minimize electrical noise.

We counted the number of eggs in each clutch (i.e., clutch size) and measured the maximum length, width, and thickness of each clutch to the nearest 0.1 mm using dial calipers while clutches rested horizontally on a flat surface, attached to their plastic cards. Then, for testing, we mounted each egg clutch in a vertical position by securely taping its plastic support card to a brick. Each test clutch was subjected to a series of standardized impacts and water drops, largely following methods for the characterization of egg-clutch mechanics in *A. callidryas* described in Caldwell (2010). Impact tests were intended to generate broad-spectrum energy in a standardized manner rather than replicate a specific natural egg-clutch disturbance, and water drops were intended to excite vibrations similar to those excited by individual raindrops falling on eggs from above. Our objective was to compare the fundamental mechanical properties of *A. callidryas* and *A. spurrelli* egg clutches that affect vibrations that embryos perceive, independent of additional variation that may be introduced by the way snakes, or other physical disturbances, interact with different types of clutches. The forcing of clutches by snakes and the vibrations it generates are clearly different and much more variable than occurs with standardized impacts. Nevertheless, the fundamental mechanics of clutch structure will inevitably affect the vibrations embryos perceive, regardless of the source of forcing. Standardized excitation tests on clutches are more tractable than snake attacks and enable direct comparisons of egg-clutch biomechanics without the additional variation introduced by snake behavior.

To perform impact tests, we built a small pendulum to apply repeatable impacts at adjustable distances from the accelerometer. The pendulum consisted of a 1.5 g lead ball crimped to the center of a 35 cm long nylon cord whose free ends were attached to the top of the pendulum frame 13.5 cm apart from each other, creating an inverted isosceles triangle with a base of 13.5 cm and two 17.5 cm sides. The lead ball was suspended 3.5 cm away from the clutch at its natural point of rest. A 10 cm high rectangular piece of thin plastic attached to the base of the pendulum served as a release tool. The lead ball rested against the top edge of the release tool and would slip free as the release tool was slowly tilted away from the clutch, continuing past its natural point of rest to impact the egg clutch (Movie S1). This allowed for highly repeatable, precise impact testing. For each clutch, we performed a series of impact tests at 1 and 2 cm above the accelerometer. For each test we released the pendulum, allowed it to impact the clutch, then caught it after the impact, allowing the clutch to vibrate freely. We repeated this impact test five times per clutch per impact distance (Movie S1). For water drop testing, we held a pipette 10 cm directly above a clutch and allowed water drops of similar size to fall onto the clutch (Movie S2). We created standardized “large” and “small” water drops using standard 7.0 mL and narrow stem 1.5 mL transfer pipettes, respectively. We repeated water drop tests five times per drop size for each clutch.

### Analysis of vibration recordings

**Fig. 2:**
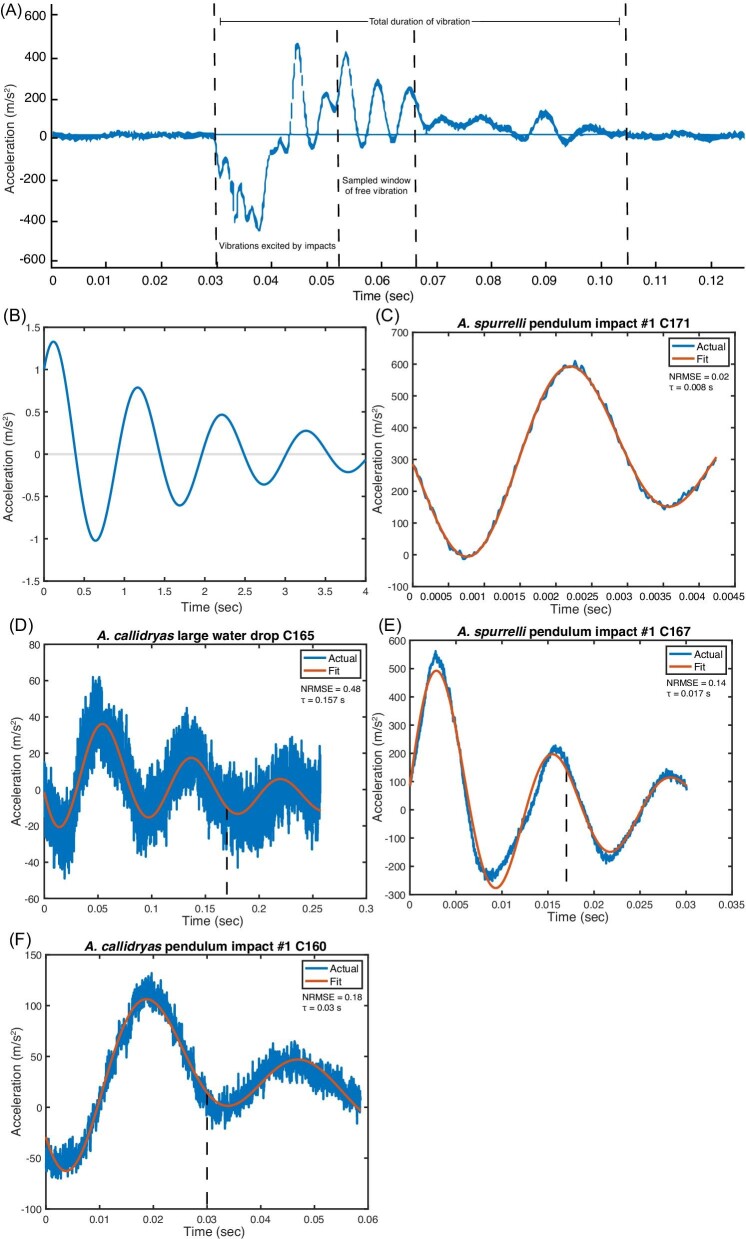
Example (**A**) waveform and windowed decaying sinusoid from excitation tests. (**B**) Acceleration computed from Equation (1) with the parameter values ω = 6 rad/s, τ = 2 s, *A*_1_ = 1 m/s^2^, and *A*_2_ = 1 m/s^2^, and *A*_3_ = *A*_4_ = *A*_5_ = 0. (**C**–**F**) Example model fits computed from Equation (1) plotted in (B). Panels **C** and **D** depict the best and worst model fits to windowed decaying sinusoids from excitation tests. The best model fit is a high amplitude vibration with a very short wavelength and the worst is a much lower amplitude vibration with a longer wavelength; the difference in noise around the sinusoid reflects scale (note axis scales differ among panels). Panels **E** and **F** are representative model fits with mean NRMSE values of pendulum impacts on *A. spurrelli* and *A. callidryas* clutches, respectively. The vertical dotted lines indicate (A) total vibration duration and periods of impact energy and sampled free vibrations and (D–F) when acceleration decayed to 36.8% of its starting value (i.e., the time constant, τ). Note that τ is outside the window in C.

We initially performed modal analysis using fast Fourier transformations (FFT) to assess the free vibration frequencies excited by our excitation tests of egg clutches. However, three aspects of our vibration recordings limited the usefulness of this approach for many recordings: (i) the initial, irregular impact energy from pendulum impact excitation tests lasted for several oscillations and affected later portions of the subsequent free vibrations, (ii) periods of free vibrations from individual excitation tests in *A. spurrelli* were short and sometimes included only one cycle of vibration, and (iii) the vibrations oscillated around a trend line with negative slope (Fig. 2A). These aspects of our recordings severely limited the sampling window of measurable free vibration for each trial and, in many cases, resulted in inaccurate estimates of free vibration frequencies from FFT, even when trials were pooled within clutches.

We therefore wrote a custom MATLAB script (available on Github at https://github.com/bguell/Comparative-egg-clutch-biomechanics) to estimate values of free vibration frequencies and rates of vibration decay in the time domain, based on the following assumptions and parameters. For the model, we first assume that the measured free vibration of the clutch is dominated by a single mode of vibration. We further assume that this mode is governed by a linear equation with viscous damping. Under these assumptions, the acceleration *a*(*t*) at any point in time obeys the following equation (Rao 2016):


(1) \begin{eqnarray*}
a(t) &=& {{A}_1}\exp ( - t/\tau )\cos (\omega t) + {{A}_2}\exp ( - t/\tau ) \sin (\omega t)\nonumber\\
&&+ {{A}_3} + {{A}_4t}+ {{A}_5}{{t}^2}\end{eqnarray*}

In this equation, ω is the frequency of vibration with dimensions of [1/t], τ is the time constant with dimensions of [t], and *A*_1_ and *A*_2_ are constants with dimensions of [L/t^2^] where L is a unit of length. The remaining constants *A*_3_–*A*_5_ simultaneously account for a quadratic trend that was observed in the data (Wu et al. 2007) while model fitting and have units of [L/t^2^], [L/t^3^], and [L/t^4^], respectively. One interpretation of the parameter τ is that it equals the time necessary for the acceleration to decay to 36.8% of its starting value (Rao 2016). Note that the period of vibration *T* is related to the frequency ω by *T* = 2π/ω. For visualization purposes, Equation (1) is plotted in Fig. 2B for sample values of the parameters.

The specific problem solved here is as follows:

- **Given** measured values of *a_n_* = *a*(*t_n_*) at times *t_n_* for *n* = 1, 2, . . ., *N*
- **Estimate** values of ω and τ

The algorithm proceeds as follows:

1. Initially estimate ω and τ by visual inspection of the measured acceleration
2. Find *A*_1_–*A*_5_ by a least-squares fit of Equation (1) to the measured values *a_n_*
3. Substitute estimates of ω, τ, *A*_1_–*A*_5_ into Equation (1) and evaluate at measurement times *t_n_* to find the estimated acceleration values $\tilde{a}$*_n_*
4. Compute the normalized root mean square error (NRMSE), ε, between the measured and estimated acceleration values according to
  \begin{eqnarray*}{\mathrm{\varepsilon }} = \sqrt {\frac{1}{N}\mathop \sum \limits_{n = 1}^N |{{a}_n} - {{{\tilde{a}}}_n}{{|}^2}}
\end{eqnarray*}
5. Improve the estimates of ω and τ using results of previous iterations
6. Return to Step 2 until ε is minimized

In the results presented here, a multidimensional unconstrained nonlinear minimization algorithm known as the Nelder-Mead simplex method (Lagarias et al. 1998) was used as implemented in the software package MATLAB.

To assess and compare the free vibration frequencies of different egg clutches, we manually sampled the largest possible windows of natural, free vibration from waveforms of vibrations excited by our tests. These windows began as soon as the waveform had a clear decaying sinusoid form (i.e., after the initial irregular, broad-spectrum vibrations excited by the impact of the pendulum or drop on the clutch) and ended when the vibrations no longer had a clear decaying sinusoidal curve and/or became indistinguishable from the noise floor of the recording (Fig. 2A). We then used these samples to compute free vibration frequencies, ω, from Equation (1) and compared them across species and excitation types. We assessed differences in vibration decay over time, between species and excitation tests, by comparing the time constant, τ (interpreted as the time necessary for the acceleration to decay to 36.8% of its starting value), computed from Equation (1).

We fit our model to 583 sampled windows that met the following criteria: (i) free vibrations from waveforms had a clear decaying sinusoidal curve, and (ii) window lengths were at least one full cycle of the curve (Fig. 2C–F). We assessed the performance of our model by calculating the NRMSE for each model fit (Ritter and Munoz-Carpena 2013). NRMSE is a frequently used measure of model accuracy and performance: It describes the difference between the predicted and observed values, allowing for evaluation of how well each model fit the data provided. Lower NRMSE values indicate better model fits. We analyzed the free vibration frequency and time constant from 521 of the sampled windows which met the following additional criteria: (i) computed NRMSE values were <0.5; (ii) computed values of the time constant, τ, were smaller than the duration of the windowed vibration sample; and (iii) computed values of free vibration frequency, ω, and the time constant, τ, were positive and within three standard deviations from the mean.

In addition to the model fitting (above), we measured vibration duration and peak amplitude from recordings in Raven Pro: Interactive Sound Analysis Software (Cornell Lab of Ornithology 2022, Version 1.6.4). We measured the total duration of each vibration in excitation tests, including both forced and free vibrations, manually from the waveforms (Fig. 2A); this includes the entire period that vibration was distinguishable above the noise floor. To estimate vibration decay over space, we also measured and compared the peak amplitude of vibrations, including both forced and free vibrations, from each pendulum impact at distances of 1 and 2 cm from the accelerometer. It was not possible to record these data blind because recordings were distinctly different between species.

### Egg-transplant snake predation experiments

**Fig. 3:**
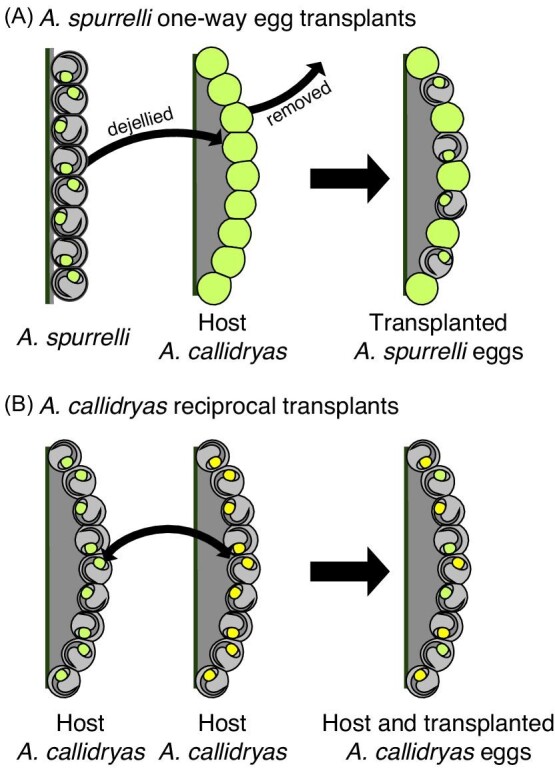
Methods for *Agalychnis* egg transplants. (**A**) Eggs of *A. spurrelli* were removed from their clutch, their tough jelly coat (dark gray outline) was removed, and each was inserted into a host *A. callidryas* clutch in place of a removed *A. callidryas* egg. Host *A. callidryas* were younger than transplanted *A. spurrelli* and not mechanoresponsive at testing (solid green). Transplanted *A. spurrelli* were mechanoresponsive at testing, but not at transplantation. (**B**) Eggs from pairs of age-matched *A. callidryas* clutches with different yolk colors were reciprocally transplanted, so each test clutch contained both host and transplanted eggs. Illustrated numbers of host and transplanted eggs do not reflect actual ratios in test clutches.

To directly assess if and how egg-clutch properties influence escape hatching in snake attacks, we compared the escape success of embryos for *A. spurrelli* eggs in their own natural clutches (nontransplanted controls) vs. those transplanted into *A. callidryas* clutches (Fig. 3A), and for *A. callidryas* eggs transplanted into a different *A. callidryas* clutch vs. those of the host clutch (Fig. 3B). We measured escape success for 28 unmanipulated *A. spurrelli* control clutches between August 3–18, 2018, using five different *L. ornata* (also reported in Güell and Warkentin 2023b). The mean ± SD daily temperature in Piro across incubation and testing days was 25.1 ± 0.6°C (measured at Osa Conservation's weather station <10 m from the ambient lab, Davis, Vantage Pro 2, 8°24ʹ14′′N, 83°20ʹ11′′W, 1.4 m altitude). We measured escape success for embryos from 29 *A. spurrelli* clutches, with each set transplanted into a different *A. callidryas* host clutch, between June 18 and July 16, 2021 (incubated and tested at mean daily temperatures of 26.6 ± 0.6°C), using four different *L. ornata*. We also reciprocally transplanted eggs between six pairs of *A. callidryas* clutches—so that each test clutch included both control (host) and transplanted eggs—and tested 11 of these mixed test clutches between June 8–25, 2019 (incubated and tested at mean daily temperatures of 23.8 ± 0.8°C), using six different *L. ornata* (*N* = 11 sibships per treatment). At our study site, *A. callidryas* embryos within clutches are typically full siblings (but see d'Orgeix and Turner 1995) while *A. spurrelli* clutches may have mixed paternity (BAG unpublished); however, based on the large population sizes, our observations of frog movements during amplexus, and spatial dispersion of the clutches we collected for these experiments, different clutches are unlikely to contain siblings.

Our goal for egg-transplant experiments was to provide *A. spurrelli* embryos with the physical environment normally experienced by *A. callidryas* embryos and measure their escape success in snake attacks in that context. To do this, we first removed 9–12 individual *A. spurrelli* eggs per clutch and manually de-jellied them, removing the tough jelly coat surrounding the vitelline membrane (Fig. 1F). In *A. callidryas* this jelly coat is softer and naturally shreds and dissipates as eggs absorb water into their perivitelline space, so at hatching embryos are enclosed only in their vitelline membrane (Pyburn 1963; Cohen et al. 2019) (Fig. 1C). We then transplanted the de-jellied *A. spurrelli* eggs into host *A. callidryas* clutches by gently inserting each *A. spurrelli* egg into the position of a previously removed *A. callidryas* egg (Fig. 3A, Movie S3).

We attempted to create the reciprocal egg-transplant test clutches for experiments—placing *A. callidryas* embryos within the physical structure of *A. spurrelli* egg clutches—but this was not feasible. Eggs in *A. spurrelli* clutches are largely held together by their tough, persistent, and sticky jelly coat (Gomez-Mestre and Warkentin 2007; Bland 2013). Because *A. callidryas* eggs transplanted into clutches of *A. spurrelli* had no adhesive coat, they fell out of the clutch, and pilot tests to add an *A. spurrelli* jelly coat to *A. callidryas* eggs for transplant were unsuccessful. Similarly, it was not possible to transplant *A. spurrelli* eggs into different *A. spurrelli* egg clutches because transplanted eggs slid out of the clutch before testing. Thus, as an alternative form of control for the egg-transplant process, we performed reciprocal egg transplantation between age-matched *A. callidryas* clutches of different colors, then conducted snake predation experiments with these mixed test clutches. Yolk color is consistent within clutches but ranges from turquoise to yellow between clutches (Warkentin et al. 2006a). To enable distinguishing transplanted from host eggs under dim light, we paired clutches with bright yellow and green yolks for reciprocal egg transplants; these are the most distinct colors, but much less common than paler green or creamy white. We performed reciprocal transplantations by gently removing 10 (*N* = 5) or 15 (*N* = 6) eggs from each age-matched *A. callidryas* clutch of different colors and inserting eggs into the positions of the previously removed eggs in the other clutch (Fig. 3B).

We conducted egg transplantations following established methods for handling individual *Agalychnis* eggs (Warkentin et al. 2017; Güell et al. 2022). We transplanted *A. spurrelli* eggs at age 3 days or early 4 days, before they became mechanoresponsive (Güell and Warkentin 2023b), to avoid hatching during transplantation. We used host *A. callidryas* clutches that were 1 or 2 days younger than the *A. spurrelli* eggs at transplantation (i.e., age 2 or 3 days), so their embryos would be unresponsive to mechanosensory cues and not hatch before or during testing (Warkentin et al. 2017; Güell and Warkentin 2023b). This allowed us to easily determine if any *A. spurrelli* hatched prior to snake attacks and accurately determine escape success in attacks (Fig. 3A). Given the rarity of easily distinguished yolk colors across the species, the color-labeling approach we used for *A. callidryas* was unfeasible combined with the added constraint of *A. spurrelli's* explosive-breeding phenology (Güell and Warkentin 2023a). However, there is no evidence that *A. callidryas* embryos use mechanosensory cues from siblings for their hatching decisions; they appear to use only predator cues (Hughey et al. 2015). We performed reciprocal transplants between *A. callidryas* clutches of different colors when embryos were 0 or 1 day old so eggs could develop within test clutches for as long as possible before testing. Neither transplantation nor age at transplantation affected embryo development, based on external morphological markers (Warkentin 2017), and we excluded any embryos with developmental abnormalities in our final count of embryos available to hatch. Test clutches were securely mounted onto bricks and kept in humidors until testing.

The mean onset of mechanosensory-cued hatching for both *A. spurrelli* and *A. callidryas* occurs at developmental stage 29 (*sensu*  Warkentin 2017), at ages 4.41 ± 0.23 and 4.68 ± 0.11 days (mean ± SD here and throughout in text), respectively, at our study site (Güell and Warkentin 2023b). *Agalychnis spurrelli* show escape-hatching responses to nocturnal snake predators on their fourth night after oviposition (i.e., one day “premature”) and their fifth night, when most embryos hatch spontaneously (Gomez-Mestre and Warkentin 2007; Güell and Warkentin 2023b). In *A. callidryas*, most spontaneous hatching at our site occurs on the sixth night after oviposition (Güell and Warkentin 2023b), and in pilot experiments on the fourth night many were still unresponsive. For both species, we conducted egg-predation experiments after embryos became mechanoresponsive and before most spontaneous hatching, on the fourth night after oviposition for *A. spurrelli* and fifth night for *A. callidryas*—i.e., one night before their peak of spontaneous hatching.

For each trial, we exposed a test clutch to attack by a cat-eyed snake, *L. ornata*, starting at 18:00 h. Snakes were collected by hand from Shampoo Pond and housed in individual mesh cages under ambient temperature, humidity, and photoperiods. Snake enclosures contained ample leaf litter, branches, and a tray of water where we placed test clutches during predation experiments. We gently placed test clutches, mounted on their bricks, in the tray filled with water to catch embryos that hatched within the snake enclosures. We checked embryos frequently (every 15–30 min) using dim red light to record any that hatched prior to snake attack (i.e., spontaneously or in response to moving their brick to the snake enclosure); these individuals were excluded from our analyses. When *A. callidryas* eggs hatched before snake attacks, we used dim white light to distinguish their yolk color. For each test clutch, we determined the number of initially transplanted eggs that were present in the clutch when attacked (*A. spurrelli*: 10.03 ± 0.68 eggs; *A. callidryas*: 10 ± 3.49; mean ± SD across 29 and 11 clutches, respectively) and the number of those that hatched during the attack. Following snake predation, we distinguished host and transplant *A. callidryas* hatchlings using yolk color, which is clearly visible through the body wall of hatchlings (Fig. 3B, Warkentin et al. 2006a).

### Mechanosensory-cued hatching of *A. spurrelli* in simulated attacks

To assess if and how the tough, rubbery egg coat of *A. spurrelli* affects embryo escape-hatching behavior and performance, we performed standardized egg-jiggling experiments (i.e., simulated attacks) on de-jellied and control eggs. We tested 210 eggs from 35 clutches between June 18 and July 15, 2021. We removed six stage-matched sibling eggs from each clutch at 3 days of age, prior to the onset of mechanoresponsiveness. We de-jellied three of them and left three with intact jelly coats, then placed each set of three siblings in their own small hexagonal weigh boat. To maintain egg hydration, we kept eggs in weigh boats in small egg humidors and misted them with rainwater frequently until testing. We began egg-jiggling tests at 18:00 h on the fourth night after oviposition, matched to the start of our snake-attack testing. For each trial, we added a drop of water to each weigh boat, then manually jiggled each set of three eggs simultaneously with a blunt metal probe for 15-s periods, alternating stimulation of control and de-jellied eggs. Thus, each egg set experienced a repeated pattern of 15 s of stimulation, 15 s of rest, for 5 min or until all the embryos hatched (see Video S1 in Warkentin et al. 2017). We recorded hatching and its latency during this period, plus any hatching that occurred during a 1-min post-stimulation observation period. Embryos that became trapped between the vitelline membrane and jelly coat (Güell and Warkentin 2023b) and required manual decapsulation after trials were scored as unhatched.

### Statistics

#### Excitation tests and vibration recordings from egg clutches

We compared clutch size, length, width, and thickness between species using Welch two-sample *t*-tests. We determined the effect of species, excitation type (i.e., four types: pendulum impacts at 1 or 2 cm from the accelerometer and small or large water drops), and their interaction on the sample window length, the NRMSE, the free vibration frequency, the vibration decay rate (i.e., time constant), the duration of vibrations, and the peak amplitude caused by our excitation tests using linear mixed models (LMMs; “lme4” package, Bates et al. 2015) followed by likelihood ratio tests of nested models. We included clutch as a random effect in all our models and used post hoc comparisons of estimated marginal means (EMMs) with Bonferroni corrections (“emmeans” package, Lenth 2023) to determine within-excitation type and within-species effects when main effects were significant. We also included clutch size (i.e., number of eggs) and clutch thickness in our initial models but found that in all cases including these predictor variables decreased the performance of our models and therefore excluded them from our final analyses. We estimated the spatial attenuation of vibration amplitude by calculating the absolute and proportional change in mean peak amplitude (taken from five repetitions per impact distance per clutch) between 1 and 2 cm away from the impact site. We then compared these values between species using Welch two-sample *t*-tests.

#### Egg-transplant snake predation experiments

We used a binomial generalized linear mixed model (GLMM) with sibship (i.e., clutch of origin) as a random effect, followed by likelihood ratio tests of nested models, to determine the effects of species, transplant treatment, and their interaction on escape-hatching success in our egg-transplant snake predation experiments; the response variable was the number of tadpoles hatched of the initial number of test embryos per clutch. We compared EMMs, as above (“emmeans” package, Lenth 2023), to determine within-species effects. Including the additional random effect of test-clutch identity (i.e., physical test structure of transplanted eggs and their host clutch) decreased model quality based on Akaike information criterion (AIC) comparisons (382.61 vs. 384.95); we therefore excluded test-clutch identity from our analysis (but see Fig. S2 for a visualization of transplant and host embryo escape rates paired by test clutches).

#### Mechanosensory-cued hatching of *A. spurrelli* in simulated attacks

We used Wilcoxon rank sum tests, with continuity corrections, to compare hatching proportion and latency of de-jellied and control eggs in egg-jiggling (simulated attack) experiments. All statistical tests were performed in the R statistical environment (R Core Development Team 2021) using RStudio (version 4.1.1; https://www.rstudio.com/products/rstudio/).

## Results

### Excitation tests and vibration recordings from egg clutches

#### Model performance

Mean egg clutch size, width, and thickness, but not length, differed between species (Table 1). The window length of samples and the NRMSE varied with species, excitation type, and their interaction (LMM, window length: main effects of species: χ^2^ = 294.78, *P* < 2.2e-16; excitation type: χ^2^ = 20.567, *P* = 0.0022; interaction: χ^2^ = 13.982, *P* = 0.003; NRMSE: main effects of species: χ^2^ = 39.079, *P* = 6.71e-08; excitation type: χ^2^ = 65.76, *P* = 3.017e-12; interaction: χ^2^ = 8.0744, *P* = 0.0445). Window length and NRMSE were lower in *A. spurrelli* than in *A. callidryas*, across excitation types (Table 2, Fig. S1). The good agreement of our linear model to measured data (Table 2, Fig. 2C–F, Fig. S1) is an implicit check that the linearity assumption of the model was valid. Note that we selected time windows to include only periods of decaying sinusoids, i.e., the hallmarks of linear and free response. We would not expect the model to fit the initial, irregular forced vibrations during and immediately following pendulum or drop impacts.

**Table 1:** Clutch size and dimensions of *A. spurrelli* and *A. callidryas* egg clutches used in excitation tests and vibration recordings at age 3 days.

	*Agalychnis callidryas* (*N* = 17)	*Agalychnis spurrelli* (*N* = 16)	*t*	*df*	*P*
Clutch size (eggs)	40.12 ± 9.79 (24–57)	33.25 ± 7.97 (20–48)	2.2154	30.392	**0.0344**
Length (mm)	46.26 ± 8.16 (32.9–59.7)	43.68 ± 6.36 (33.4–55.4)	1.0203	29.994	0.3157
Width (mm)	23.94 ± 4.27 (16.4–30.9)	20.40 ± 3.41 (16.0–28.3)	2.6378	30.223	**0.0131**
Thickness (mm)	9.42 ± 1.26 (7.2–12.4)	7.78 ± 1.12 (5.8–10.1)	3.9847	30.9	**0.0004**

Data are presented as mean ± SD (range). Significant *P*-values (<0.05) are in bold

**Table 2:** Window length of decaying sinusoids used to fit Equation (1) and computed NRMSE per species per excitation test.

Species	Excitation test	Window length (s)	NRSME
*Agalychnis spurrelli*			
	Pendulum impact at 1 cm	0.036 ± 0.022	0.13 ± 0.06
	Pendulum impact at 2 cm	0.028 ± 0.019	0.15 ± 0.09
	Large water drops	0.018 ± 0.012	0.16 ± 0.07
	Small water drops	0.017 ± 0.007	0.18 ± 0.09
*Agalychnis callidryas*			
	Pendulum impact at 1 cm	0.137 ± 0.075	0.14 ± 0.05
	Pendulum impact at 2 cm	0.160 ± 0.102	0.21 ± 0.10
	Large water drops	0.147 ± 0.108	0.20 ± 0.08
	Small water drops	0.16 ± 0.115	0.24 ± 0.08

Data are presented as mean ± SD.

#### Model estimations of free vibration frequency and the time constant of amplitude decay

**Fig. 4:**
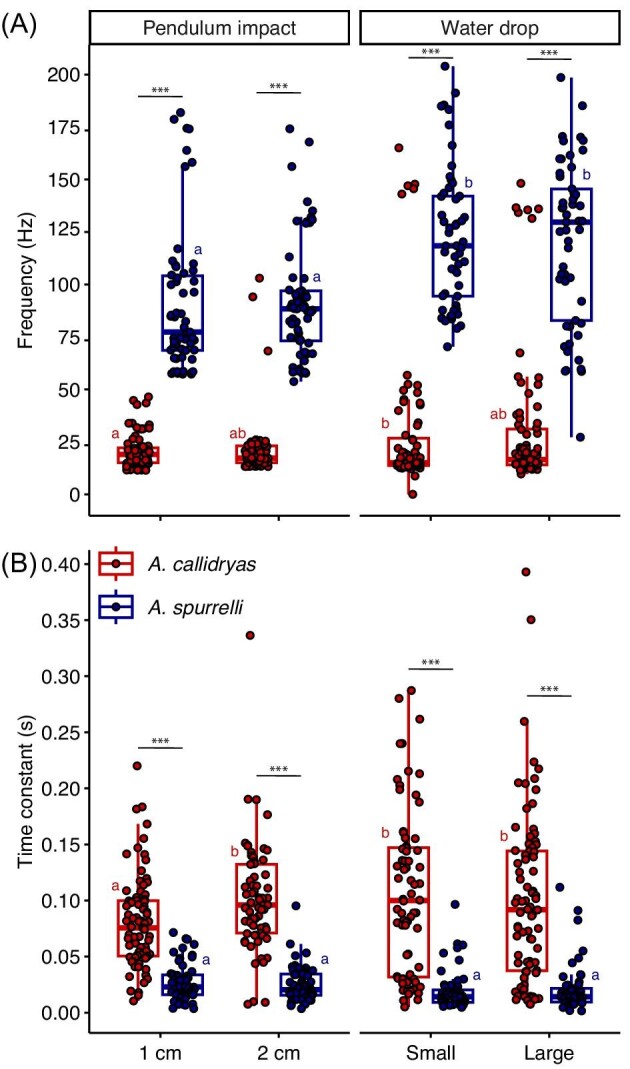
(**A**) Dominant free vibration frequency, ω, and (**B**) time constant, τ, of amplitude decay computed from Equation (1) using windowed decaying sinusoids excited by pendulum and water drop impacts on *A. spurrelli* and *A. callidryas* clutches at age 3 days. Data points are jittered horizontally and represent individual excitation tests. Results of within excitation type post hoc analyses from mixed models are shown in black: ns *P* > 0.05, * *P* < 0.05, ** *P* < 0.01, *** *P* < 0.001. Different letters indicate significant differences between excitation types from post hoc comparisons within species. Box plots show medians, interquartile range (IQR), and extent of data to ± 1.5 × IQR.

The free vibration frequencies of egg clutches varied with species, excitation type, and their interaction (LMM, main effects of species: χ^2^ = 514.28, *P* < 2.2e-16; excitation type: χ^2^ = 74.58, *P* = 4.683e-14; interaction: χ^2^ = 18.289, *P* = 0.0004; Fig. 4A). Frequencies differed between species within every excitation type (all pairwise comparisons *P* < 0.0001) and were about four times higher in *A. spurrelli* egg clutches than in *A. callidryas* (105.15 ± 36.7 Hz vs. 25.65 ± 26.06 Hz, mean ± SD across excitation types, Fig. 4A). Post hoc analysis revealed that the significant species × excitation type interaction was due to lower frequencies excited by pendulum impacts in *A. spurrelli* which were on average ∼30 Hz lower than those excited by water drops (all pairwise comparisons between pendulum impacts and water drops *P* < 0.0001). In contrast, pendulum impacts excited frequencies only ∼9 Hz lower than those excited by water drops in *A. callidryas*; only frequencies from pendulum impacts at 1 cm and small water drops were different from each other (*P* = 0.0301, all other pairwise comparisons *P* > 0.05).

We found a significant effect of species, excitation type, and a species × excitation type interaction on the time constant, τ—i.e., the rate of vibration decay, interpreted as the time for vibrations to decay to 36.8% of their starting value (LMM, main effects of species: χ^2^ = 255.13, *P* < 2.2e-16; excitation type: χ^2^ = 24.433, *P* = 0.0004; interaction: χ^2^ = 14.147, *P* = 0.0027, Fig. 4B). The time constant differed between species within every excitation type (all pairwise comparisons *P* < 0.0001) and was about five times shorter in *A. spurrelli* than in *A. callidryas* (0.02 ± 0.02 s vs. 0.10 ± 0.06 s, mean ± SD across excitation type, Fig. 4B). The significant species × excitation type interaction was a result of a difference in the time constant across excitation types in *A. callidryas*, but no difference in *A. spurrelli* (all pairwise comparisons *P* ≥ 0.7965). Specifically, the time constant was shorter for pendulum impacts at 1 cm from the accelerometer compared to all other excitation types in *A. callidryas* (all pairwise comparisons *P* ≤ 0.0015).

#### Manual measurements of peak amplitude and vibration duration

**Fig. 5:**
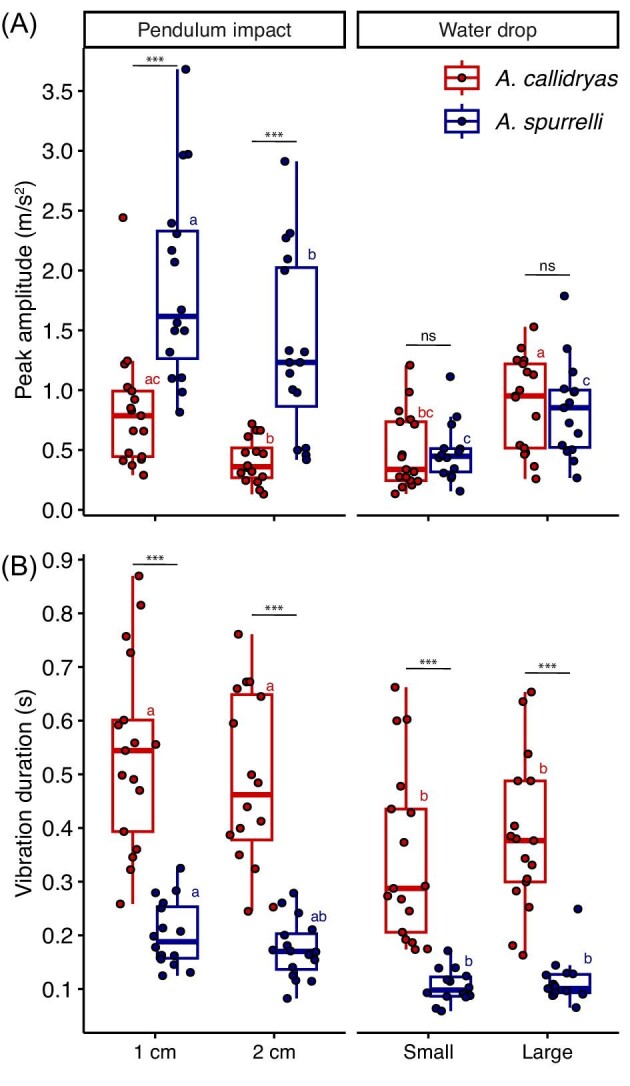
(**A**) Peak amplitude and (**B**) total duration of vibrations excited by pendulum and water drop impacts, measured from accelerometers within *A. spurrelli* and *A. callidryas* clutches at age 3 days. Data points are jittered horizontally and are the mean values per clutch. Results of within excitation type hoc analyses from mixed models are shown in black: ns *P* > 0.05, * *P* < 0.05, ** *P* < 0.01, *** *P* < 0.001. Different letters indicate significant differences between excitation types from post hoc comparisons within species. Box plots show medians, IQR, and extent of data to ± 1.5 × IQR.

Species, excitation type, and their interaction all had a significant effect on peak amplitude (LMM, main effects of species: χ^2^ = 59.464, *P* = 3.76e-12; excitation type: χ^2^ = 69.055, *P* = 6.387e-13; interaction: χ^2^ = 35.349, *P* = 1.028e-07, Fig. 5A). The significant species × excitation type interaction was caused by higher peak amplitudes from pendulum impacts on *A. spurrelli* clutches compared to *A. callidryas* ones (both pairwise comparisons *P* < 0.001), but similar peak amplitudes across species from both large and small water drops (both pairwise comparisons *P* = 0.962, Fig. 5A). Peak amplitude was consistently higher from pendulum impacts closer to the accelerometer and from larger water drops (Fig. 5A). Post hoc analyses showed that peak amplitude differed across all excitation types in *A. spurrelli*, except between water drop tests (*P* = 0.05, all other pairwise comparisons *P* ≤ 0.0067). In *A. callidryas*, peak amplitude from impacts at 1 cm from the accelerometer and large water drops were similar to each other (*P* = 0.6811), but both were different from impacts at 2 cm from the accelerometer (both pairwise comparisons *P* ≤ 0.0213).

The total duration of vibrations caused by our excitation tests (measured as in Fig. 2A) was on average about one third as long in *A. spurrelli* clutches than in *A. callidryas* clutches (0.15 ± 0.06 s vs. 0.44 ± 0.18 s, mean ± SD across excitation type; LMM, main effect of species: χ^2^ = 136.96, *P* < 2.2e-16, Fig. 5B). Vibrations caused by pendulum impacts lasted longer than those excited by water drops (LMM, main effect of excitation type: χ^2^ = 42.988, *P* = 1.173e-07). There was no significant species × excitation type interaction effect (χ^2^ = 4.64, *P* = 0.2).

**Fig. 6:**
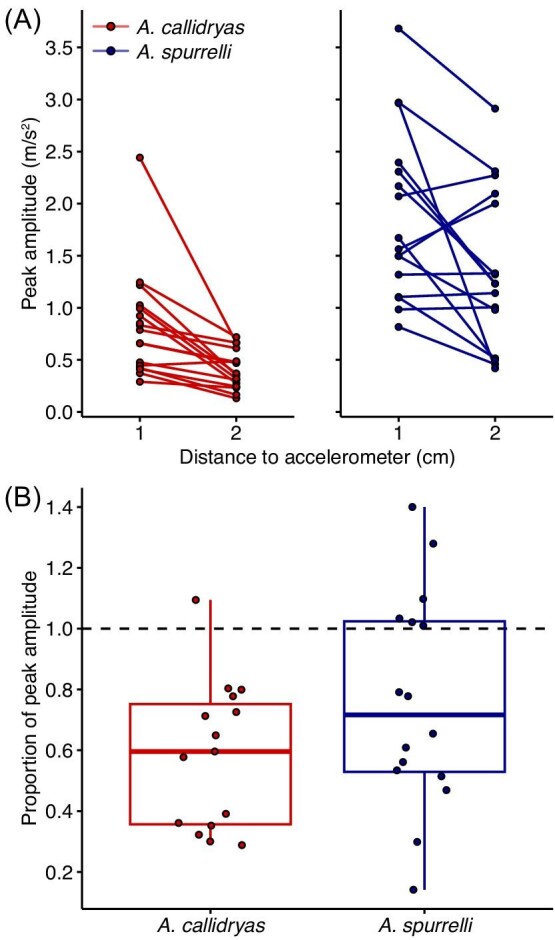
Visualization of spatial attenuation (or enhancement) of peak vibration amplitude in pendulum impact tests on *A. spurrelli* and *A. callidryas* clutches at age 3 days. Lines in (**A**) connect mean measured amplitudes for impacts 1 and 2 cm away from the accelerometer in the same clutch. (**B**) Amplitudes measured for impacts 2 cm away in proportion to those 1 cm away from the accelerometer. Points above the dashed line (1 on the y-axis) indicate that peak amplitude measured was higher for impacts farther from the accelerometer within a clutch. Box plots show medians, IQR, and extent of data to ± 1.5 × IQR; data points represent individual clutches (mean of five tests at each distance) and are jittered horizontally in (B).

For most egg clutches, pendulum impacts showed lower peak amplitude at greater distance from the impact site (Fig. 6A). However, the peak amplitude measured 2 cm from the impact site was higher than at 1 cm away for 1 of 17 *A. callidryas* clutches and 6 of 16 *A. spurrelli* clutches (Fig. 6). The spatial rate of vibration decay, measured as either the absolute or proportional change in peak amplitude between 1 and 2 cm away from the impact site, was not significantly different between species, regardless of whether we excluded clutches for which measurements of peak amplitude were larger at the greater distance (absolute change: *t* = –0.94771, *df* = 18.79, *P* = 0.3553; proportional change: *t =* –1.6567, *df* = 26.578, *P* = 0.1093, Fig. 6).

### Egg-transplant snake predation experiments

**Fig. 7:**
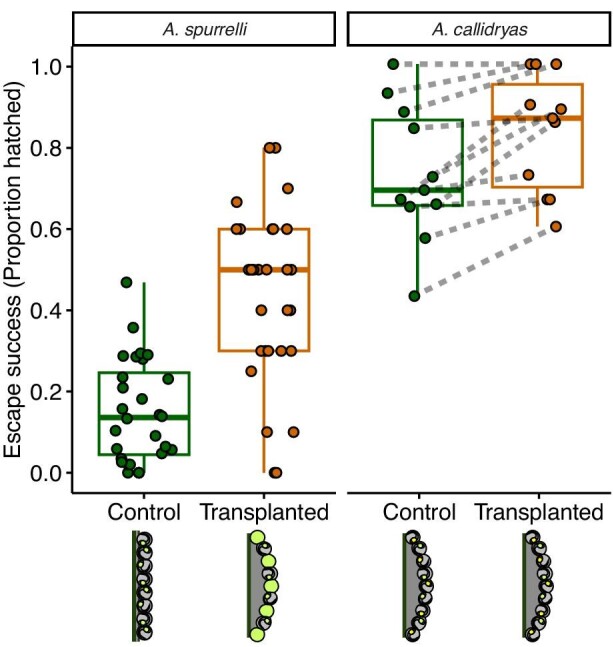
Escape-hatching success of control (green) and transplanted (orange) *A. spurrelli* and *A. callidryas* embryos in attacks by cat-eyed snakes, *L. ornata*, 1 day before their peak of spontaneous hatching. Solid green eggs in graphic represent younger (unresponsive) host-clutch *A. callidryas* eggs. Control *A. spurrelli* embryos were not transplanted; control *A. callidryas* were transplant hosts. Data points are jittered horizontally and represent escape success of a group of siblings tested together in each treatment. Dashed lines connect data from *A. callidryas* siblings tested in their original clutch (control) and after transplantation. Box plots show medians, IQR, and extent of data to ± 1.5 × IQR.

Escape-hatching success in snake attacks varied with species, transplant treatment, and their interaction (GLMM, main effects of species: χ^2^ = 52.917, *P* = 3.23e-12; transplant treatment: χ^2^ = 33.124, *P* = 6.416e-08; interaction effect: χ^2^ = 5.3423, *P* = 0.02081). Post hoc analysis revealed that escape success was substantially higher for transplanted *A. spurrelli* embryos compared to nontransplanted controls (44 ± 21% vs. 15 ± 13%; *P* < 0.0001, Fig. 7). In contrast, transplanting *A. callidryas* eggs into age-matched conspecific clutches had a much smaller effect on escape-hatching success in attacks (83 ± 15% vs. 73 ± 17%, *P* = 0.0256, Fig 7).

### Mechanosensory-cued hatching of *A. spurrelli* in simulated attacks

**Fig. 8:**
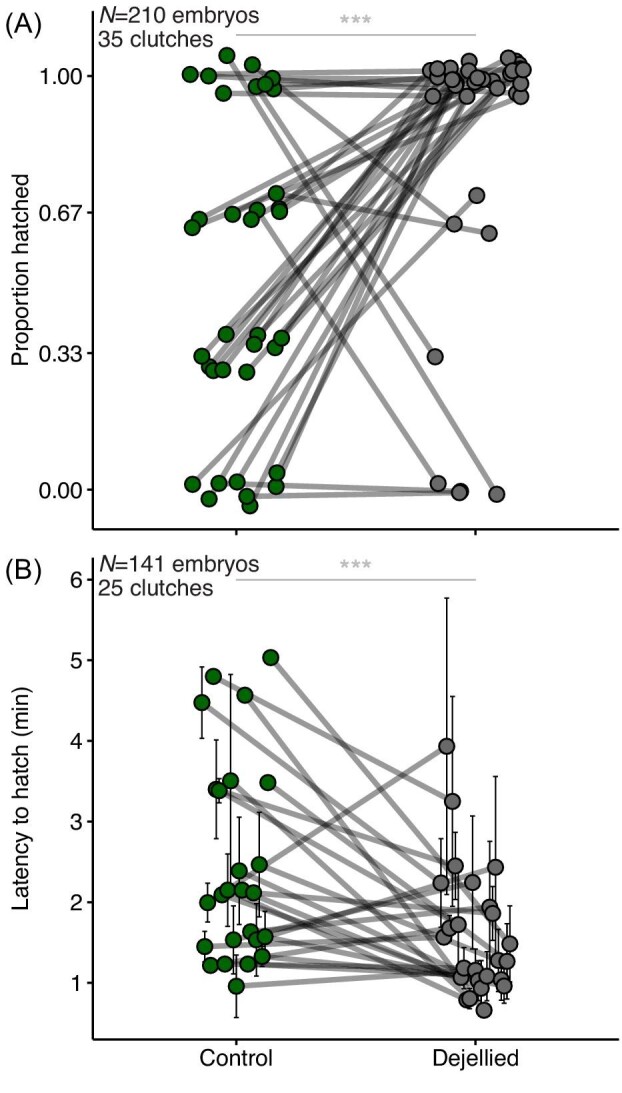
Proportion of embryos hatched (**A**) and latency to hatch (**B**) from control and de-jellied *A. spurrelli* eggs in response to mechanosensory (egg-jiggling) cues at age 4 days. (A) Data points represent proportion hatched of the three siblings tested per clutch per treatment and are jittered horizontally and vertically; lines connect siblings across treatments. (B) Data points show mean latency to hatch (±SE) for siblings in each treatment and are jittered horizontally; lines connect siblings that hatched across treatments. Embryos that did not hatch are not shown. For some clutches only one egg (of the three tested) hatched in a treatment resulting in no error bars for some points in B. *** *P* < 0.001.

In egg-jiggling trials with *A. spurrelli*, a larger proportion of embryos attempted to hatch, and a larger proportion successfully hatched, from de-jellied vs. sibling control eggs (attempts: 83.8% vs. 64.8%*, W* = 377, *P* = 0.0022; successful hatching: 83.8% vs. 50.5%, *W* = 305.6*, P* < 0.0001; Fig. 8A). The 14.3% of control embryos (*N* = 15 of 105) that attempted to hatch but did not succeed all emerged partway through their vitelline membrane but remained trapped within their jelly coat and were unable to escape; this never happened to de-jellied eggs. No embryos fully hatched after becoming trapped within their eggs. All but 4 of 35 sibships had higher (or equal) hatching response rates in de-jellied eggs compared to control eggs; embryos from 2 sibships did not hatch in either treatment. Moreover, in five of the clutches tested, no control embryos hatched, while all their siblings in de-jellied eggs hatched. Latency to hatch of siblings across treatments was shorter from de-jellied eggs than controls (1.58 ± 1.1 vs. 2.35 ± 1.3 min; *W* = 2452.5, *P* = 5.074e-05, Fig. 8B).

## Discussion

The differences in structure and vibration properties of *A. spurrelli* and *A. callidryas* egg clutches clearly affect the escape-hatching response of their embryos. In contrast to *A. callidryas, A. spurrelli* lay eggs in thin monolayers, so clutches lack a gelatinous core, and each egg is covered with a tough jelly coat. Transplanting de-jellied *A. spurrelli* embryos into *A. callidryas* clutches significantly increased their escape success compared to age-matched, nontransplanted conspecifics attacked within their natural egg clutches. We also found that *A. spurrelli* embryos removed from their tough jelly coat were more likely to hatch and hatched faster in response to simulated attack (egg-jiggling) cues compared to their sibling controls. Together our results suggest that whole egg-clutch and individual egg-capsule structure decrease the information available to embryos and impair their hatching performance. The difference in egg clutch structure affected vibration mechanics largely as predicted; *A. spurrelli* clutches had higher free vibration frequencies and faster vibration damping than did *A. callidryas* clutches. Lastly, our work serves as a proof of concept for using nontraditional methods of modal analysis in the time domain to analyze the biomechanical properties of complex and ecologically relevant structures to better understand how animals respond to substrate-borne vibration cues.

### Egg-clutch structure affects vibration biomechanics

**Fig. 9:**
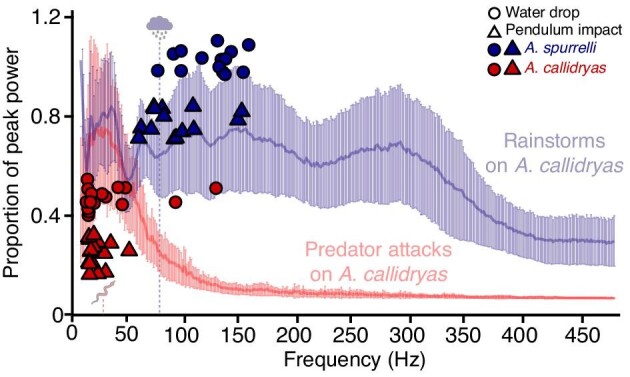
Free vibration frequencies excited by pendulum (triangle) and water drop (circle) impacts on *A. spurrelli* (blue data points) and *A. callidryas* (red data points) clutches at age 3 days mapped on the average relative distribution of energy across frequencies for vibrations in *A. callidryas* egg clutches during predator attacks and rainstorms, from Caldwell et al. (2010). X-axis position of data points represents clutch means across impact distances and water drop sizes; vertical positions are arbitrary. Power spectra are means across recorded clutch disturbances (rainstorms, *N* = 19 clutches; predators include snakes: *L. ornata* (formerly *L. septentrionalis*), *N* = 11; *L. rhombifera* (formerly *L. annulata*), *N* = 17; *L. ahaetulla, N* = 13; wasp: *Polybia rejecta, N* = 18), standardized to peak power, and 95% confidence intervals, reproduced from Caldwell et al. (2010). The mean dominant frequencies across predator attacks and of rainstorms recorded in *A. callidryas* egg clutches are indicated on the figure with a snake and rain cloud illustration.

The free vibrations of any structure will depend on its physical properties. Thinner and stiffer structures are expected to deform less in disturbances and to resonate at higher free vibration frequencies (Snowdon 1968). We predicted that *A. spurrelli* egg clutches would therefore have higher free vibration frequencies compared to the more gelatinous, thicker egg clutches of *A. callidryas*. The free vibration energy in all our tested *A. spurrelli* egg clutches was well over 50 Hz, while most *A. callidryas* clutches oscillated at much lower frequencies, supporting our prediction. The free vibration frequencies we found for *A. callidryas* clutches (25.7 ± 26.1 Hz, *N* = 296 mean ± SD across all excitation types) were similar to modal vibration frequencies previously measured for this species (water drops: 23.5 ± 13.7 Hz, *N* = 10; pendulum impacts and plucks: 19.8 ± 7.7 Hz, *N* = 25) (Caldwell 2010) and mostly below 50 Hz, in the range that elicits the strongest hatching response in vibration playbacks (Caldwell et al. 2009). This range includes the dominant frequencies in snake attacks [*L. ornata* (formerly *L. septentrionalis*), 19.0 ± 13.7 Hz, *N* = 11; *L. rhombifera* (formerly *L. annulata*), 26.3 ± 15.2 Hz, *N* = 17; *Leptophis ahaetulla*, 38.4 ± 24.1 Hz, *N* = 13; Fig. 9] (Caldwell et al. 2009). Whether clutch motion in snake attacks is dominated by free vibrations *per se*, or by forced vibrations from snake movements that share these frequencies, the dominance of low frequencies in snake attacks is important for *A. callidryas* escape-hatching response (Caldwell et al. 2009). We have not recorded vibrations from *A. spurrelli* clutches during snake attacks. However, the consistency between frequencies excited by snake attacks and our pendulum impacts in *A. callidryas* clutches, and the higher frequencies excited by pendulum impacts in *A. spurrelli*, suggest that snake attacks likely excite higher frequencies in *A. spurrelli* clutches as well. The free vibration frequency of *A. spurrelli* was four times higher than the dominant frequencies of snake attacks, and even higher than the dominant frequency of rainstorms, recorded from *A. callidryas* clutches (*A. spurrelli*: 105.2 ± 36.7 Hz, *N* = 225 vs. rainstorms: 77.7 ± 99.8 Hz, *N* = 19; Fig. 9) (Caldwell et al. 2009). If *A. spurrelli* share similar frequency-based hatching decision rules as *A. callidryas*—where embryos hatch in response to very low vibration frequencies but considerably less to higher frequencies—then higher vibration frequencies could partially explain the lower hatching response of *A. spurrelli* to snake attacks. These results motivate vibration recordings of snake attacks and rain in *A. spurrelli* egg clutches and vibration playback experiments to determine risk assessment and hatching decisions in response to specific vibration properties.

Several aspects of the temporal pattern of vibrations—such as pulse durations and interpulse intervals—affect hatching in *A. callidryas* (Warkentin 2005; Warkentin et al. 2006b). However, unlike vibration frequencies, the temporal properties of vibrations with different effects are not clearly matched to patterns in natural disturbances, and indeed the patterns in natural disturbances are difficult to quantify. Measured temporal patterns from vibrations in snake attacks differ from those that elicit the most hatching in *A. callidryas* in playback experiments (Warkentin 2005; Warkentin et al. 2006b). As snakes bite and pull at eggs they often create closely spaced vibrations similar to those created by hard rain, making the distinction between risk cues and benign stimuli challenging for embryos (Warkentin 2005). However, unlike hard rain, snake attacks also include long vibration-free periods (e.g., as they chew and swallow eggs without contacting the clutch). These long gaps help *A. callidryas* embryos effectively distinguish vibrations caused by snake attacks and heavy rain (Jung et al. 2022). Both the rate of vibration decay (i.e., the time constant) and the total duration of vibrations from our excitation tests were substantially shorter in *A. spurrelli* clutches (Figs. 4B, 5A). Shorter vibration durations and faster vibration damping in *A. spurrelli* suggest that the temporal pattern of vibrations in snake attacks and rainstorms is also different in their clutches. Determining whether the biomechanical properties of *A. spurrelli* clutches make it harder for embryos to distinguish between predator cues and benign sources of information will require further investigation.

We expected that the reduced clutch jelly and stiff structure of *A. spurrelli* egg clutches would inhibit the propagation of vibrations to embryos throughout the clutch, thus limiting the distance over which embryos receive vibrational cues. The fact that peak amplitude of pendulum impacts at 2 cm away from the accelerometer was lower than at 1 cm is consistent with the decay of vibrations through space. However, we found no interspecific differences in amplitude loss over space: The change in peak amplitude caused by our pendulum impacts at 1 and 2 cm away from the accelerometer did not differ between species (Fig. 6). We were instead surprised to find that some values of peak amplitude measured at 2 cm from the impact site were higher than those at 1 cm, suggesting a nonlinear and unpredictable change of vibration strength over space. Vibrations do not always decrease monotonically in amplitude with distance, particularly through certain complex substrates such as plants (Michelsen et al. 1982; Fahy and Gardonio 2007). This is likely because natural substrates are often structurally complex and spatially variable, with subcomponents that vary in biomechanically relevant properties, causing them to have more variable damping properties and show stronger frequency dependence of damping than do more uniform media (Michelsen et al. 1982; Markl 1983). The physical environment in which an *Agalychnis* embryo experiences vibrations is complex. Each embryo is surrounded by perivitelline fluid, within a vitelline membrane and, for *A. spurrelli*, a jelly coat that imposes turgor pressure (Fig. 1). Eggs are packed together, often contacting six other eggs. *Agalychnis spurrelli* eggs are closely adhered to their oviposition substrate while in well-hydrated *A. callidryas* the gelatinous jelly core of the clutch separates many eggs from the substrate (Fig. 1). The presence of fluid in egg clutches, i.e., the perivitelline fluid, may alter the free vibration of the systems, but it does not change the presence of fixed natural frequencies and decay rates of the structures. It is well known that systems having coupled fluid and solid elements have fixed natural frequencies and temporal decay rates (Junger and Feit 1986). Nevertheless, how vibrations propagated through the egg clutches in our experiment may have also been influenced by several factors other than the stiffness of the whole structure, including the spatial variation in biomechanical properties of the clutch, standing waves, reflections within the clutch, clutch size, and subtle differences in egg clutch hydration. Despite our consistent methods for excitation testing and recording vibrations within clutches, slight variations in the relative position of the accelerometer within clutches and the exact impact site may have also affected vibration propagation.

### Custom MATLAB script for analyzing the biomechanics of complex biological structures in the time domain

Our investigation of effects of egg-clutch structure on vibration biomechanics depended on our ability to record vibrations from natural egg clutches and estimate their vibration properties. The methods we used to record vibrations in egg clutches are well established in *A. callidryas* (Warkentin 2005; Caldwell et al. 2009; Caldwell 2010; Warkentin et al. 2022) and were easily adapted to *A. spurrelli* clutches. However, analyzing the vibration recordings was more challenging. We expected that the gelatinous structure of *A. callidryas* egg clutches and the fact that they readily stretch and deform in physical disturbances would affect their damping and free vibration properties. Specifically, these clutch characteristics suggest that they are highly damped and that natural free vibrations are likely nonlinear, limiting the use of traditional modal analyses in the frequency domain to determine clutch vibration properties (e.g., amplitudes, frequencies, damping, etc.). Our custom MATLAB script allowed us to estimate the free vibration frequency and rate of vibration decay for each clutch in the time domain using very short sampling windows from individual excitation tests. This was useful since vibrations in *A. spurrelli* egg clutches were very short (Figs 2 and 5). Moreover, this approach also allowed us to account for the gradual negative slope of free vibrations excited by our excitation tests. Future studies aiming to analyze the biomechanical properties of ecologically relevant structures may also find these alternative analytical approaches helpful.

### Egg and clutch properties impair escape hatching in *A. spurrelli*

Whole clutch and individual egg properties affected the escape-hatching response to real snakes and simulated attacks in *A. spurrelli*. Transplanted *A. spurrelli* eggs that were attacked by snakes within the physical structure of a gelatinous *A. callidryas* clutch had nearly three-fold higher escape success than nontransplanted controls (Fig. 7), and de-jellied eggs hatched more often and more quickly in simulated attacks (Fig. 8). The mean escape success of our control *A. spurrelli* clutches in snake attacks was only slightly higher than that measured in the early 1990s from a population at a nearby site in Corcovado National Park (15% vs. 9%; Gomez-Mestre and Warkentin 2007). We may have captured more of the natural variation in escape rates for 4-day-old *A. spurrelli* embryos in our experiments since we tested over three times the number of clutches as Gomez-Mestre and Warkentin (2007) (*N* = 28 and 8). It is also possible that the increase in temperatures on the Osa Peninsula since the early 1990s (mean increase of 0.18°C per decade, NOAA National Centers for Environmental Information 2022) has accelerated rates of embryo development causing our 4-day-old embryos to show greater hatching responses, characteristic of more advanced embryos. The fact that some *A. callidryas* clutches in our pilot tests were mechanoresponsive at age 4 days, while they were mechanoresponsive only at 5 days in the early 1990s in Corcovado (Warkentin 1995), is consistent with this interpretation. Nevertheless, both measurements of escape rates in *A. spurrelli* are significantly lower compared to those of *A. callidryas*. The consistent measurements of low escape success in natural *A. spurrelli* clutches across field sites and studies over 25 years apart suggest that these escape rates represent their species-typical hatching responses to snakes.

The substantial increase in escape-hatching success of embryos transplanted into *A. callidryas* clutches suggests that egg clutch and/or egg capsule structure contribute to *A. spurrelli's* low escape-hatching success in snake attacks. However, our inability to transplant eggs into *A. spurrelli* clutches limits our ability to precisely distinguish effects of transplantation from those of clutch structure. We specifically could not assess if egg transplantation affected *A. spurrelli* and *A. callidryas* differently. Transplantation into conspecific clutches does not change the structure of egg capsules or clutches, and we often remove individual eggs of both species for experiments with no apparent effect on development or subsequent behavioral responses to physical disturbance cues (Warkentin et al. 2017; Güell et al. 2022; Güell and Warkentin 2023b). Our method for *A. spurrelli* one-way transplants into *A. callidryas* clutches did not affect the subsequent development of embryos within host clutches based on external morphological markers (Warkentin 2017), nor was it likely to affect future behavioral responses to physical disturbance cues not perceivable at the time of transplantation. The fact that the working memory of *A. callidryas* embryos, and presumably those of *A. spurrelli*, is only about 45 s (Jung et al. 2022) is also consistent with this assumption. It did, however, fundamentally change the whole egg-clutch and individual egg-capsule structure of transplanted embryos, presumably making vibrations created by snakes more threatening and escaping through de-jellied eggs easier. Mean escape success also increased significantly in transplanted *A. callidryas* eggs. Nonetheless, escape rates for transplanted *A. callidryas* eggs were within the range of host (control) eggs, and escape rates for both transplanted and host eggs were consistent with rates we found previously for 5-day-old *A. callidryas* embryos at our site (83 ± 15% and 73 ± 17% compared to 78.2 ± 11.6%, Güell and Warkentin 2023b). This is in striking contrast to the effect of transplanting *A. spurrelli* eggs into *A. callidryas* clutches, where transplanted embryos had a mean escape rate (44%) nearly outside the range of escape rates for control eggs (0–47%). Thus, while transplantation *per se* appeared to slightly increase embryo escape success in our experiments, the much larger increase in escape success of *A. spurrelli* eggs transplanted into *A. callidryas* clutches, compared to *A. callidryas* eggs transplanted into conspecific clutches, is consistent with a key role of egg and clutch structure in determining escape-hatching success in *A. spurrelli* embryos.

The mean temperature across incubation and testing days of our egg-transplant snake predation experiment varied from 23.8 ± 0.8°C during reciprocal *A. callidryas* trials to 25.1 ± 0.6°C and 26.6 ± 0.6°C during *A. spurrelli* control and transplant trials, respectively. This difference was largely due to conducting experiments in different years. *Agalychnis* embryos raised at 29°C reach mechanoresponsiveness about 10 h sooner than those raised at ambient temperatures of 26°C and 50 h sooner than those raised at 22°C (Güell and Warkentin 2018). However, a 1.5°C higher rearing temperature and its effects on embryo development cannot explain the 30% increase in escape success of transplanted *A. spurrelli* embryos compared to nontransplanted controls (Fig. 7). Mean escape rates of *A. spurrelli* clutches never surpass 28% (Gomez-Mestre and Warkentin 2007; Güell and Warkentin 2023b), even at age 5 days when embryos are more developed than our 4-day-old test subjects and most hatch spontaneously. This suggests that variation in rearing and testing temperatures between *A. spurrelli* transplants and controls is unlikely to explain the observed differences in escape success.

Variation in risk assessment, the hatching process, or both can influence the overall latency to hatch of embryos (Güell et al. 2022). Our simulated attack (egg-jiggling) experiment suggests that the tough external layer of jelly in *A. spurrelli* eggs affects both of these processes. De-jellied eggs were more likely to hatch than their control siblings (85 vs. 51%, Fig. 8A). However, 14% of embryos in control eggs experienced hatching complications, emerging part way through the vitelline membrane but remaining stuck within an intact jelly capsule after attempting to hatch. A larger proportion of de-jellied embryos attempting to hatch (85 vs. 65%) is consistent with an effect of the jelly coat on risk assessment. One plausible explanation is that the tough jelly coat of *A. spurrelli* has mechanical properties that limit the vibration information available to embryos within eggs, essentially acting as a vibration-damping structure. If so, de-jellying *A. spurrelli* eggs may have altered the stimulation that embryos received during attacks, affecting their hatching responses in a second way, independent of whole clutch vibration mechanics. Our finding of higher escape success in transplanted *A. spurrelli* embryos (Fig. 7) may reflect effects of both egg de-jellying and overall clutch structure (Fig. 8). However, the increase in hatching response of *A. spurrelli* eggs transplanted into *A. callidryas* clutches was much greater than that of de-jellied vs. control eggs (2.9 vs. 1.3 times higher) suggesting that de-jellying alone cannot explain the former.

The persistent jelly coat also seems to influence the hatching process in *A. spurrelli*. Although all embryos that experienced hatching complications in this study required rescue, we have also observed temporary, nonlethal cases of hatching complications—where embryos eventually successfully exited their eggs—earlier in development at the onset of hatching responses to hypoxia and simulated attack (egg-jiggling) cues (Güell and Warkentin 2023b). Hatching complications also occur in *A. callidryas*, although they are less common—embryos can become displaced from their initial rupture site and recover by repositioning themselves or creating new holes in their eggs (Salazar-Nicholls et al. 2017). Clearly these are different kinds of complications, with different risks and effects on the hatching process. However, both can increase the hatching latency of embryos that manage to successfully exit their eggs. The common observation of hatching complications across development and contexts in *A. spurrelli* suggests that their low escape-hatching success in snake attacks may be, in part, due to the effect of egg-capsule structure on hatching performance (i.e., hatching speed). Given that snakes can consume entire *Agalychnis* clutches in just a few minutes (Warkentin et al. 2007, BAG personal observation from videos), the difference we found in mean hatching latency of 0.77 min between de-jellied and control *A. spurrelli* eggs could be the difference between escaping vs. being eaten in an attack. Evaluation of the frequency of these hatching complications and their impact on hatching effectiveness across risk contexts and development would be informative.

Vibration-cued hatching offers an excellent opportunity to study how parentally produced structures affect embryos’ ability to use incidental cues to inform their behavior. The mechanosensory information available to embryos that develop within gelatinous egg masses, egg cases, or other structures that physically hold eggs will depend on the vibration mechanics of these structures. Clearly, egg-clutch and individual egg-capsule structure are important factors that contribute to *A. spurrelli's* low escape-hatching success. However, escape rates of *A. spurrelli* eggs transplanted into *A. callidryas* clutches were not as high as control *A. callidryas* eggs, and simulated attack cues did not elicit complete hatching in de-jellied *A. spurrelli* eggs. Thus, our understanding of why *A. spurrelli* embryos show such low escape rates in snake attacks is clearly incomplete. It is possible that *A. spurrelli* has evolved different behavioral decision rules than *A. callidryas*, matched to the vibration biomechanics of its own clutch structure. Unlike other substrates that transmit behaviorally relevant vibrational information, egg-clutch structure and oviposition site choice are subject to selection on parents (Delia et al. 2020). Thus, interspecific variation in other, perhaps stronger, selective factors may have led to the evolution of different clutch phenotypes, generating pleiotropic effects on escape-hatching responses. The present study motivates additional research, including vibration recordings of snake attacks and rain on *A. spurrelli* clutches paired with vibration playback experiments to determine how these embryos use vibrational information for risk assessment in escape-hatching decisions.

## Supplementary Material

obae006_Supplemental_Files

## Data Availability

The data collected for and analyzed in this paper and the MATLAB script and associated files are available on Github at https://github.com/bguell/Comparative-egg-clutch-biomechanics.
