# Supplementary material for: Egg-Clutch Biomechanics Affect Escape-Hatching Behavior and Performance
Source: Integr Org Biol. 2024 Mar 13;6(1):obae006. doi: 10.1093/iob/obae006 (PMC10995723; doi:10.1093/iob/obae006)
Supplement: obae006_Supplemental_Files [file obae006_supplemental_files.zip › Figure S2.pdf]

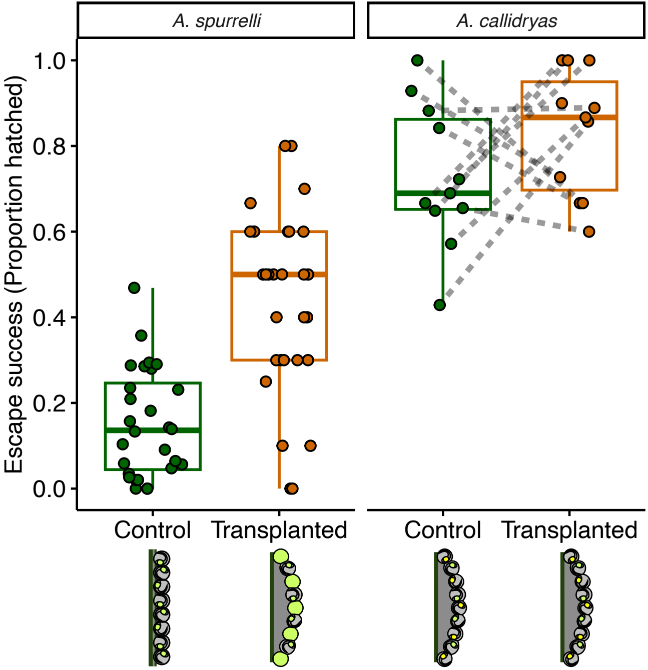
A. spurrelli
A. callidryas
Escape success (Proportion hatched)
1.0
0.8
0.6
0.4
0.2
0.0
Control
Transplanted
Control
Transplanted
