# Supplementary figures and images for: Egg-Clutch Biomechanics Affect Escape-Hatching Behavior and Performance
Source: Integr Org Biol. 2024 Mar 13;6(1):obae006. doi: 10.1093/iob/obae006 (PMC10995723; doi:10.1093/iob/obae006)

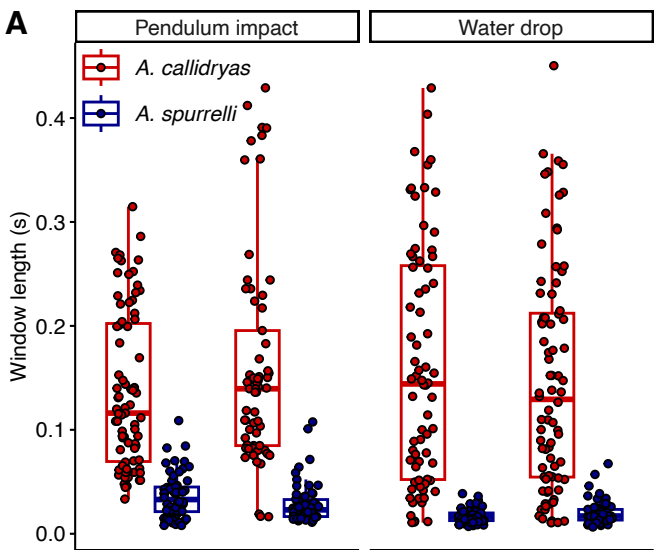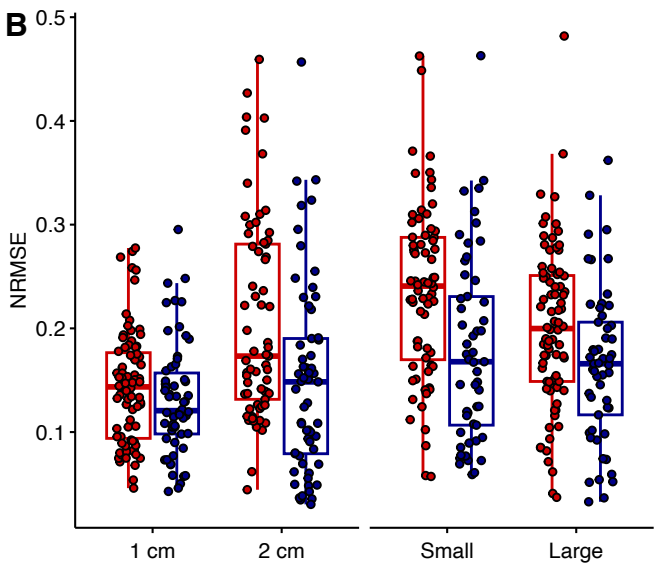

Supplement: obae006_Supplemental_Files [file obae006_supplemental_files.zip › Figure S1.pdf]
